# The Prediction of the Gas Utilization Ratio Based on TS Fuzzy Neural Network and Particle Swarm Optimization

**DOI:** 10.3390/s18020625

**Published:** 2018-02-20

**Authors:** Sen Zhang, Haihe Jiang, Yixin Yin, Wendong Xiao, Baoyong Zhao

**Affiliations:** 1 School of Automation and Electrical Engineering, University of Science and Technology Beijing, Beijing 100083, China; g20158511@xs.ustb.edu.cn (H.J.); yyx@ies.ustb.edu.cn (Y.Y.); wdxiao@ustb.edu.cn (W.X.); zhby@ustb.edu.cn (B.Z.); 2 Key Laboratory of Knowledge Automation for Industrial Processes of Ministry of Education, School of Automation and Electrical Engineering, University of Science and Technology Beijing, Beijing 100083, China

**Keywords:** data-driven model, gas utilization ratio, TS fuzzy neural network (TS-FNN), particle swarm optimization (PSO) algorithm, blast furnace (BF)

## Abstract

Gas utilization ratio (GUR) is an important indicator that is used to evaluate the energy consumption of blast furnaces (BFs). Currently, the existing methods cannot predict the GUR accurately. In this paper, we present a novel data-driven model for predicting the GUR. The proposed approach utilized both the TS fuzzy neural network (TS-FNN) and the particle swarm algorithm (PSO) to predict the GUR. The particle swarm algorithm (PSO) is applied to optimize the parameters of the TS-FNN in order to decrease the error caused by the inaccurate initial parameter. This paper also applied the box graph (Box-plot) method to eliminate the abnormal value of the raw data during the data preprocessing. This method can deal with the data which does not obey the normal distribution which is caused by the complex industrial environments. The prediction results demonstrate that the optimization model based on PSO and the TS-FNN approach achieves higher prediction accuracy compared with the TS-FNN model and SVM model and the proposed approach can accurately predict the GUR of the blast furnace, providing an effective way for the on-line blast furnace distribution control.

## 1. Introduction

The blast furnace (BF) ironmaking process is the high energy-consuming process [1,2,3], producing high levels of environmental pollution which is becoming an increasingly seriously problem nowadays and making it necessary to devise energy-saving and consumption-reduction methods for iron and steel production [4], especially for BF ironmaking. At present, researchers all over the world a mainly focusing on the coke ratio prediction of the blast furnace because it is an important economic indicator during the BF ironmaking process. However, the coke ratio is the energy-consumption indicator for the short period (such as one day), and its prediction mainly relies on the judgment and evaluation by the operators in the factory. The gas utilization ratio (GUR) represents the ratio of the carbon monoxide to the carbon dioxide in the BF. It is a key parameter to measure the degree of the gas-solid-phase reduction reaction of the BF ironmaking. It is also a key factor to characterize the energy consumption which can directly evaluate the energy utilization of a BF [5]. GUR can thus express the current blast furnace operation situation and the energy consumption in time.

Researchers have carried out some research on the GUR. For the calculation mechanism of the GUR, Lahm [6] created a joint calculation method and obtained a GUR calculation formula; Na et al. [7] improved the formula considering the impact of the BF ironmaking materials. Xiang et al. [8] studied the BF ironmaking mechanism and combined it with the actual production situation. They studied the correlation between the GUR and the other indicators that indicate the production efficiency. Based on a theoretical derivation and field experience, Wei et al. [9] established a linear regression equation of the GUR based on the relevant parameters which can optimize the production. Zhou et al. [10] proposed a new calculation method of the GUR and worked on GUR theory. Hu et al. [11] proposed a comprehensive strategy of improving the blast furnace burden structure, according to the blast furnace production status, by adjusting the coordination between the upper and the lower parts of the blast furnace. 

However, research on the GUR prediction nowadays is only concentrated at the mechanism level [12]. Actually BF smelting is a highly complicated production process. It is hard to reflect the complex BF smelting process with mechanism modeling and it is difficult to establish the inherent hidden relationships among the variables. The mechanism models are mainly based on the smelting mechanism, expert experience and some statistical methods. This leads to inaccurate scheduling, serious waste of the resources and low efficiency of the BF production. Data-driven models can more accurately predict the GUR. In this paper, the GUR is chosen as the indicator of the BF energy-consumption. The TS-FNN and particle swarm optimization (PSO) methods are used to establish the prediction model according to the field data. The algorithm adopts the PSO to optimize the parameters of the TS model. In order to ensure the accuracy of the model, this paper firstly analyzes the factors that affect the GUR. We choose box-plots to eliminate the abnormal values of the raw data. Then, we use the wavelet de-noising method to de-noise the data. The results show that the model can accurately predict the GUR, laying the foundation for the steady production of the BF.

The remaining parts of this paper are arranged as follows: Section 2 gives some preliminaries, including the analysis of the influencing factors of the GUR and the data preprocessing of the raw data. Section 3 proposed the algorithm to analyze the correlation between the parameters of the BF and the GUR. Section 4 presents the GUR prediction model based on TS-FNN. Section 5 presents the PSO-TS-FNN prediction model using real data. Section 6 presents a performance comparison of different models. Conclusions are given in Section 7.

## 2. Analysis of the Relevant Factors of the GUR and the Data Preprocessing

The BF ironmaking process mainly involves the complex physical changes and the chemical reactions such as the oxidation reaction. Many factors affect the GUR in these chemical reactions. If all related factors are used as the prediction model inputs, some unnecessary redundancy will inevitably lead to low model performance and accuracy. Therefore, the primary task of the prediction modeling is to determine the relevant factors of the GUR. That means we have to find out what parameters are the key factors which are closely relevant to the GUR.

### 2.1. Selection of the Input Parameters of the Prediction Model

GUR ($\eta_{co}$) can be calculated by the content of the gas CO and ${\mathrm{CO}}_{2}$ in the top gas of the BF; the calculation formula is shown in Equation (1):

(1) $$\eta_{\mathrm{co}}=\frac{\left({\mathrm{co}}_{2}\right)}{\left({\mathrm{co}}_{2}\right)+\left(\mathrm{co}\right)}$$

where $\left({\mathrm{co}}_{2}\right)$ is the volume content of carbon dioxide ${\mathrm{CO}}_{2}$ in the top gas of the BF; (CO) is the volume content of carbon monoxide CO in the top gas of the BF. There are two forms of reduction of the iron ore in a blast furnace. One is the direct reduction and the other is the indirect reduction. The corresponding reaction formulas are shown in Equations (2) and (3), respectively:

(2) FeO+C=Fe+CO

(3) $$\mathrm{FeO}+\mathrm{nCO}=\mathrm{Fe}+{\mathrm{CO}}_{2}+(n-1)\mathrm{CO}$$

where, CO is carbon monoxide, ${\mathrm{CO}}_{2}$ is carbon dioxide, Fe is elemental iron, FeO is the ferric oxide, C is carbon. 

This paper mainly considers the influence of the operation parameters of the blast furnace. A reasonable and efficient blast furnace iron making system can guarantee a high GUR and a low coke ratio. Increasing the air temperature and pressure can provide sufficient temperature and pressure so as to effectively promote the chemical reduction reactions in the BF. The air speed influences the activity of the BF hearth which in turn determines the size of the combustion zone and the gas flow distribution. The increase of the top pressure and the top temperature in the BF can effectively promote the rate of the chemical reactions in the BF. Therefore, the input parameters of the prediction model are initially selected as the air temperature, the air pressure, the top pressure, the oxygen enrichment, and the top temperature of the BF. The specific analysis of each factor follows.

### 2.2. The Rejection of the Outliers of the Raw Data of the BF

In the process of data-driven modeling, the outliers of the raw data will seriously affect the modeling accuracy. Therefore, we firstly analyze the distribution characteristics of the raw data. We proposed the appropriate outlier detection method to eliminate the outliers. At present, the researchers usually use 3*σ* rule or *Z* fraction method to discard the outliers.

The 3*σ* rule or the *Z* fractional method assumes that the data obeys the normal distribution. In probability statistics, as long as the amount of data is large enough, all the large data will eventually obey the normal distribution. However, there are only around 1000 sets of data in this paper, so the amount of data is small. If there is an outlier, the mean and standard deviation will be greatly affected. 

We firstly make a statistical analysis of the 6 types of the BF variables i.e., the blast temperature, the blast pressure, the top pressure, the oxygen enrichment, the top temperature, and the gas utilization ratio. We will use normal distribution histogram and normal probability graph to judge whether they are normal distribution. 

**Figure 1:**
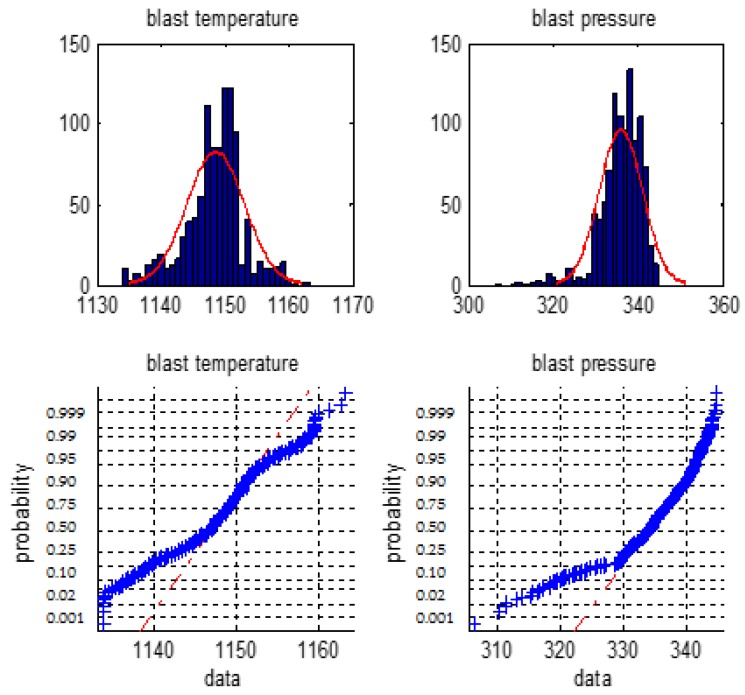
The distribution histogram and the normal probability plot of the blast temperature and the blast pressure.

**Figure 2:**
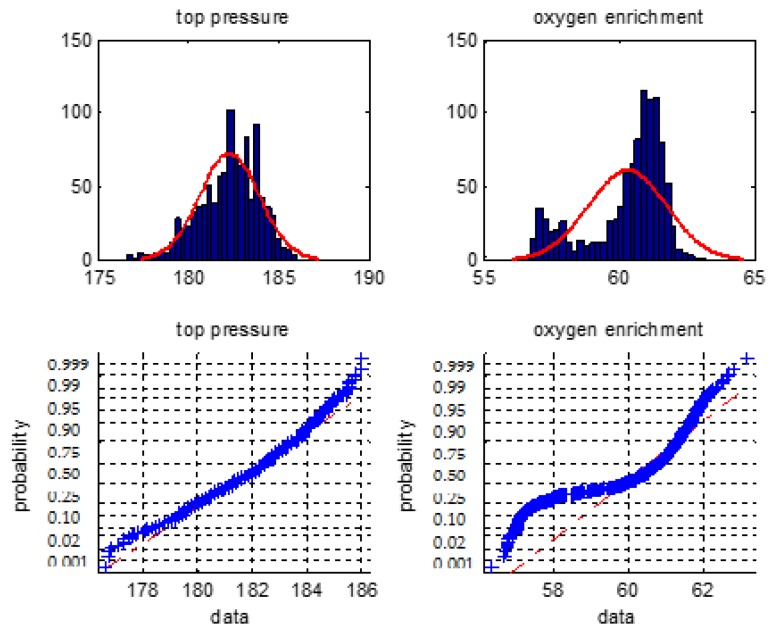
The distribution histogram and the normal probability plot of the top pressure and the oxygen enrichment percentage.

**Figure 3:**
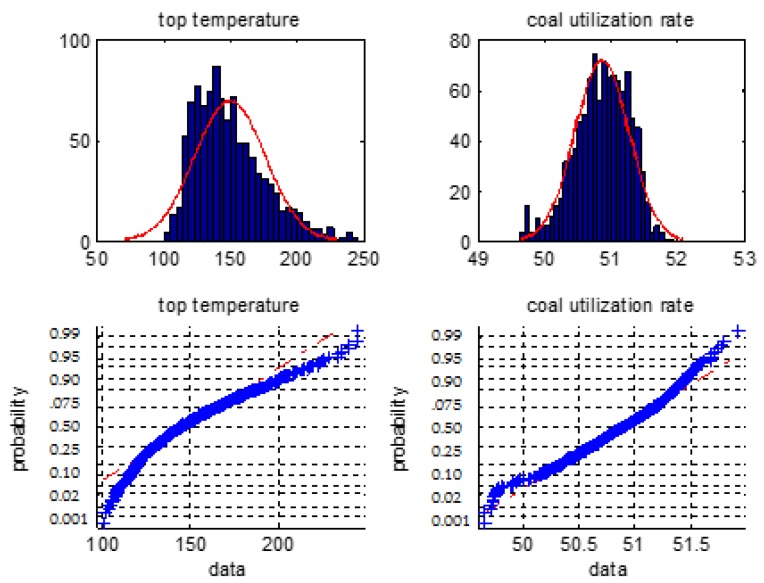
The distribution histogram and the normal probability plot of the top temperature and the coal utilization ratio.

Figure 1, Figure 2 and Figure 3 are normal distribution histogram and normal probability graph of the six classes of variables. If they have a normal distribution, their normal curves are red lines. We observed that the six classes of variables do not show a normal distribution, so we cannot use the 3*σ* rule or *Z* fractional method to remove the outliers.

In this paper, we propose using the box graph method to eliminate the outliers. It is a statistics method suitable for data which does not obey a normal distribution. Box-plots [13] are also known as box drawings. They are a statistical graph used to display data dispersion. They are named this way because their shape is similar to a box. The details of box-plots can be seen in [13]. The data collected from a real iron and steel factory will be processed by this method below. 

**Figure 4:**
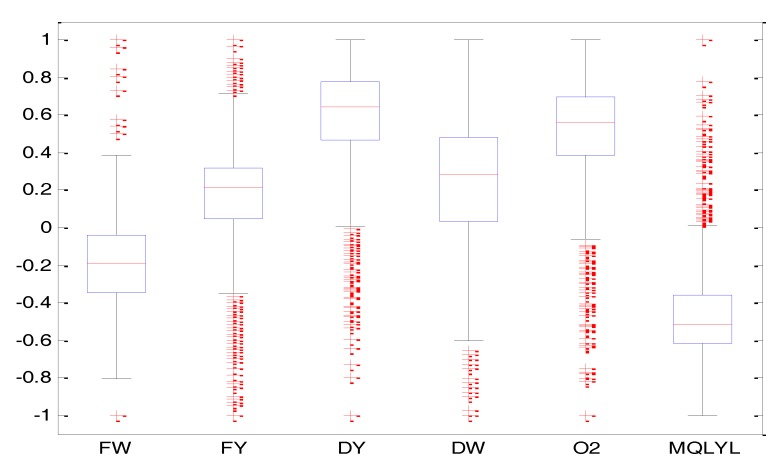
The box diagram of each parameter.

We collected the data from the blast furnace site. We choose 1400 groups of real data to be processed. We drew the box-plot for each parameter of the blast furnace. The results can be seen in Figure 4. In this figure, FW represents the blast temperature, FY is the blast pressure, DY is the top pressure, DW is the top temperature, O_2_ is the enrichment percentage, MQLYL represents the GUR. The red crossing marks indicate extreme outliers. In this paper, the total number of outliers is 200 groups. This paper only removes the extreme outliers for the prediction.

## 3. The Parameter Correlation Analysis

In order to find the correlation between the inputs and the outputs of the data-driven model, a large number of references have been reviewed. A lot of researchers use the Pearson correlation coefficient (PCC) or Spearman correlation coefficient (SCC) to estimate the correlation of their data. However, the PCC or the SCC can only calculate a linear correlation of the data (thus not being very useful for nonlinear data). BF iron-making is a nonlinear and strongly coupled process, so it is not reasonable to use a linear correlation coefficient to analyze the correlation of its data. In our research, we use the mutual information principle to analyze the correlation of the data from the BF. 

### 3.1. Mutual Information Principle and the Generalized Correlation Coefficient

According to the principles of information theory [14,15], the uncertainty of the off-line stochastic variable *X* can be expressed by the information entropy $H_{X}$:

(4) $$H\left(X\right)=-\overset{q}{\underset{i=1}{\sum }}P\left(x_{i}\right)\mathrm{lg}P\left(x_{i}\right)$$

where, $P\left(x_{i}\right)$ is the probability of $x_{i}$; q is the total number of the events (States) that might occur. Obviously, for fully determined variables *X*, H(X)=0; for random variables *X*, H(X)>0 (non-negative).

For two different random variables *X* and *Y*, the conditional entropy of *X* for *Y* can be defined as H(X/Y):

(5) $$H\left(X/Y\right)=-\sum_{i,j=1}^{q}P\left(y_{j}\right)P\left(x_{i}|y_{j}\right)\mathrm{lg}P\left(x_{i}|y_{j}\right)$$

where, $P\left(y_{j}\right)$ is a probability event of $y_{j}$, $P\left(x_{i}|y_{j}\right)$ is the conditional probability of the event $x_{i}$ under the condition of $y_{j}$.

Obviously, when *X* and *Y* are completely independent, H(X/Y)=H(X). When *X* and *Y* are completely related (a fully determined relationship), H(X/Y)=0. For general dependent variables, H(X/Y)>0. Similarly, we can get H(X/Y) by the conditional entropy of *Y* for *X*.

For event *X*, its entropy decreases with the existence of event *Y* and the correlation between them, namely the mutual information I(X,Y). It is defined as follows:

(6) I(X,Y)=H(X)−H(X/Y)

It can be proved that the mutual information is non-negative *I*(*X*,*Y*), and it also has mutual property, that is:

(7) I(X,Y)=H(X)−H(X/Y)=H(Y)−H(Y/X)=I(Y,X)

The federated information of *X* and *Y*,  H(X,Y) is introduced as:

(8) $$H\left(X,Y\right)=-\underset{i,j}{\sum }P\left(x_{i},y_{j}\right)\mathrm{lg}P\left(x_{i},y_{j}\right)$$

where, $P\left(x_{i},y_{j}\right)$ is the joint probability of $x_{i}$ and $y_{j}$, i.e., the probability that the event $x_{i}$ will occur simultaneously with the event $y_{j}$. Such mutual information *I*(*X*,*Y*) is:

(9) I(X,Y)=H(X)+H(Y)−H(X,Y)

It is worth pointing out that the mutual information does not have any special requirement for the type of the variable distribution. We gave the mutual information results of the real data from the blast furnace in Table 1. 

In mutual information [16], if I(X,Y)>δI(Y,Y) and δ=0.5, we consider that there is a strong correlation between the parameter *X* and *Y*. From the above table, we can observe that the operation parameters have a strong correlation with the GUR, so we use the six parameters in Table 1 to build the data-driven model.

### 3.2. The Wavelet De-Noising

In the process of BF production, the signal contains a lot of noise which is usually not white noise. The traditional Fourier transform can only processes the data in the frequency domain rather than the time domain. Wavelet analysis can analyze the signal in time-frequency domain and it can effectively eliminate the mutation so as to realize the de-noising of the non-stationary signals. 

The soft threshold method is applied to de-noise the raw data in the section. The wavelet basis function is selected as Demy. In general, the decomposition scale of wavelet decomposition is set to 4 in one-dimensional decomposition. The formula for calculating the threshold λ is as follows:

(10) $$\lambda =0.3936+0.1829\log_{2}N$$

where *N* is the number of the wavelet coefficients obtained by the wavelet decomposition on the current scale for the noisy signals.

**Figure 5:**
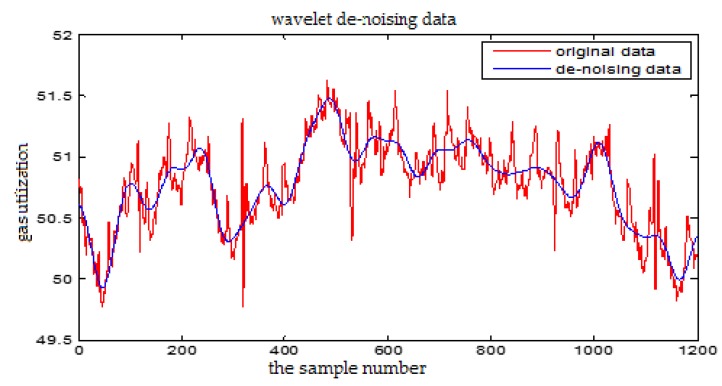
The wavelet de-noising for the Gas Utilization Rate

**Figure 6:**
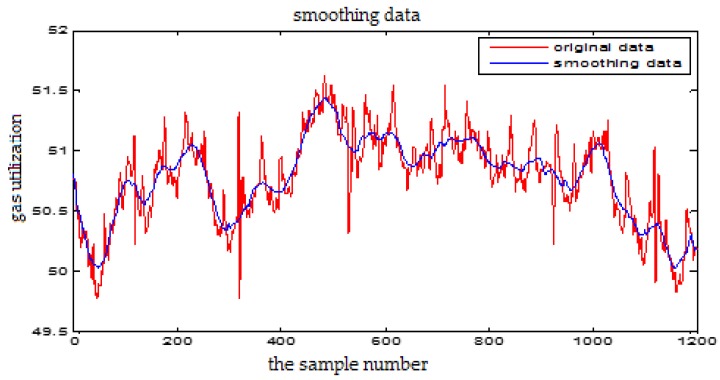
The Fourier transform de-noising for the Gas Utilization Rate

From Figure 5 and Figure 6, we can see that the high frequency noise can be filtered well, but it will cause partial amplitude distortion. The wavelet de-noising can keep the signal characteristics well, effectively remove the spikes and burrs in the data, and eliminate the strong oscillation noise. So we choose wavelet de-noising method in this paper.

## 4. The Prediction Model of the GUR Based on the T-S Model 

GUR is affected by many factors. GUR is a variable with strong coupling and serious nonlinear relationship with many factors. We combined the fuzzy technology and the neural network technology to construct the fuzzy neural network (FNN). FNN can automatically process the fuzzy information. It has the advantages of the fuzzy logic system and the neural network. Its convergence speed is very fast and the approximation performance is outstanding which has attracted the interest and attention of many researchers. 

In the fuzzy system, there are two main methods to express the fuzzy model. One is fuzzy neural network based on Mamdha fuzzy rule, and the other one is FNN [17] based on T-S model. We adopted TS-FNN in this paper. TS-FNN is defined in the following “if-then” rule form.

If $x_{1}$ is $A_{1}^{i}$, $x_{2}$ is $A_{2}^{i}$, $\cdots ,\;x_{k}$ is $A_{k}^{i}$:

(11) $$y_{i}=p_{0}^{i}+p_{1}^{i}x_{1}+\cdot \cdot \cdot +p_{k}^{i}x_{k}$$

where $A_{j}^{i}$ is a fuzzy set, $p_{j}^{i}\;(j=1,2,\;\cdots ,\;k)$ is the fuzzy parameters.

Set the input variable x= [$x_{1}$,$x_{2}$,$\cdots ,x_{k}$], the membership degree of each input variable $x_{j}$ is calculated according to the fuzzy rule:

(12) $$\mu_{A_{j}^{i}}=\exp(-\left(x_{j}-c_{j}^{i}{)}^{2}\right)\;j=1,2,\ldots ,n,\;i=1,2,\ldots ,n$$

In this formula, the center of the membership function is $c_{j}^{i}$. k is the number of the arguments. The number of fuzzy subsets is n. The fuzzy calculation method is used to calculate the membership degree. The multiplication operator is used on the fuzzy operator:

(13) $$\omega_{i}=\mu_{A_{j}^{1}}*\mu_{A_{j}^{2}}\;(x_{2})\;*\cdots *\mu_{A_{j}^{k}}\;(x_{k})\;i=1,2,\cdots ,n$$

where “*” means multiplication.

The output value $y_{i}$ of the fuzzy model can be obtained from the fuzzy calculation:

(14) $$y_{i}=\overset{n}{\underset{i=1}{\sum }}\omega_{i}(p_{0}^{i}+p_{1}^{i}x_{1}+L+p_{k}^{i}x_{k})/\overset{n}{\underset{i=1}{\sum }}\omega^{i}$$

**Figure 7:**
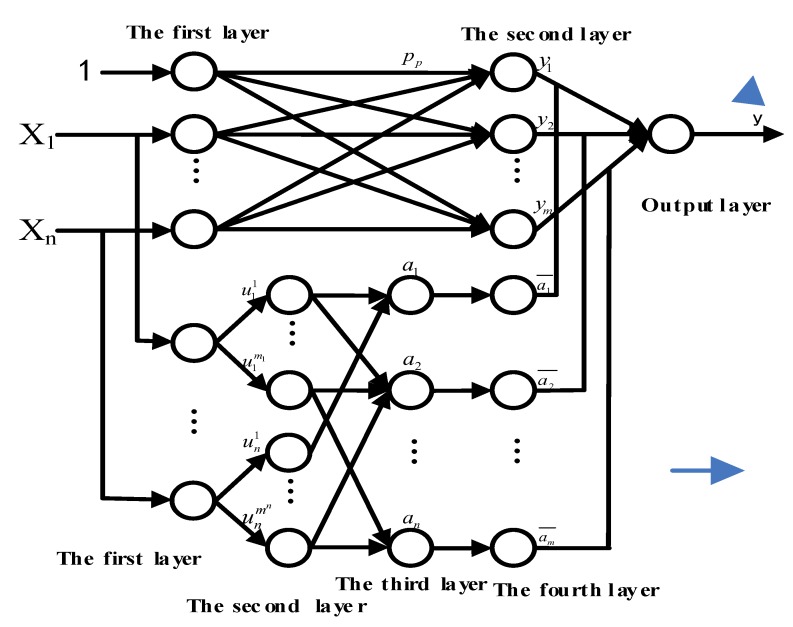
The structure of the T-S fuzzy neural network.

According to the fuzzy rules discussed above, we can construct the TS-FNN [18]. This is shown in Figure 7. The network mainly consists of two parts. One is used to match the antecedent network of the fuzzy rule antecedent, and the latter is used to match the posterior part of the fuzzy rule. The specific meaning of each layer of the network will be described below and the node function corresponding to each layer is given in detail. 

(1) The antecedent network

The first layer is the input layer and the beginning of the network. It is the direct connection of each component of the input vector $x_{i}$. The number of nodes is equal to the number of input vectors. 

The second layer is the fuzzy layer, also known as the intermediate layer. In this layer, each node represents a language variable value. The main function of this layer is to blur the input data. Its function is to calculate the membership function of each input component belonging to each linguistic variable value. 

The third layer is the fuzzy rule layer. It is also the middle layer. It is used to match the antecedent of the fuzzy rules. Its node represents each fuzzy rule. And the fitness of each rule can be calculated. 

The fourth layer is the intermediate layer, which is mainly used to achieve normalization. 

(2) Posterior network

The first layer of the posterior network is the same as the antecedent network, i.e., the input layer, and it provides a constant for the posterior part of the fuzzy rule. 

The second layer is the middle layer, and each node of it represents a rule, which can be used to get the posterior parts of each rule.

The third layer is the output layer, which is designed to calculate the output of the system. 

It can be seen that we can finally get the output *Y* after a series of calculation. The weighted coefficient can be obtained by calculating the normalized fitness function of each fuzzy rule, and the connection weight is obtained through the intermediate layer in the antecedent network.

This paper uses the actual January 2016 data of a BF located at the Anshan Iron and Steel Company. The sampling interval is 1 min. We selected 1400 sets of data, of which 1200 sets are used in the algorithm after removing out the outliers. According to the rules of the data model, the number of training data is 3–5 times more than the testing data. We selected 1000 of them as the training data, and the remaining 200 groups are used as the testing data of the model. 

The input variables are blast pressure, blast temperature, top temperature, top pressure, oxygen enrichment, and the output is GUR. The simulation results of the proposed algorithm are as follows.

**Figure 8:**
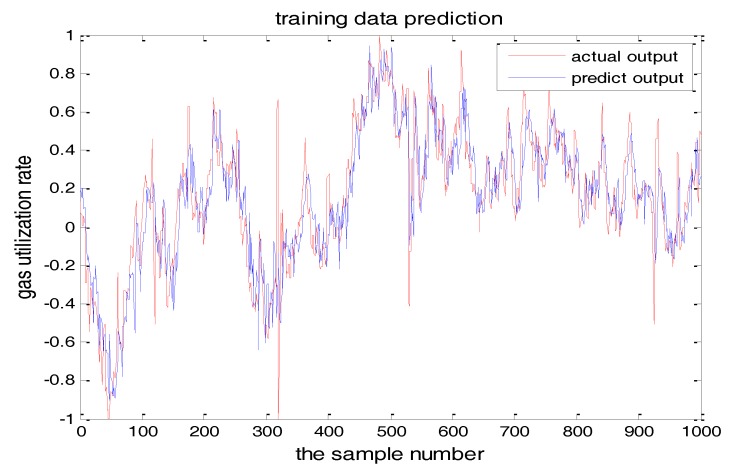
TS-FNN training result when the number of hidden nodes is 11.

**Figure 9:**
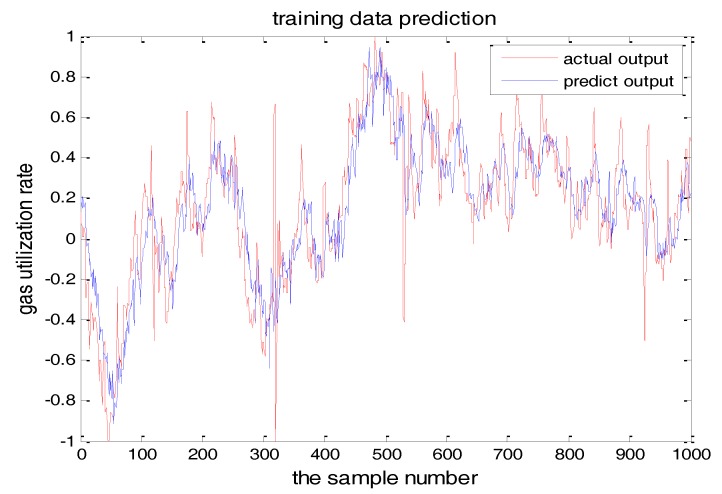
The TS-FNN training result when the number of hidden nodes is 7.

**Figure 10:**
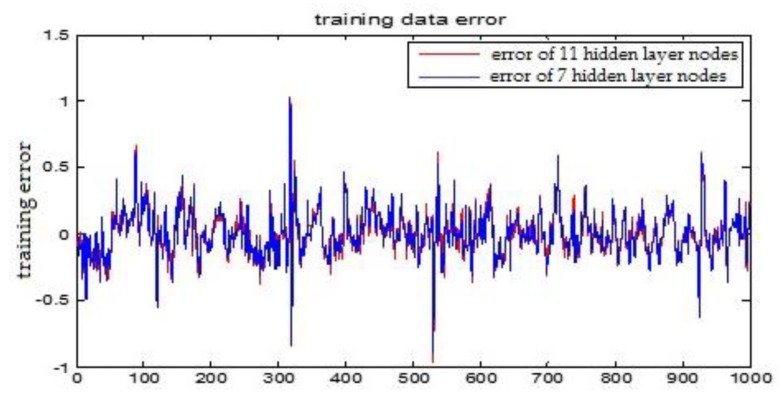
The TS-FNN training error curve when the number of hidden layer nodes is 7 and 11, respectively.

The number of hidden layer nodes in T-S-FNN will have a great impact on the output. Figure 8 and Figure 9 showed the simulation results of 1000 groups of training data, in which the number of hidden nodes is 11 and 7, respectively. The other parameters of the network are the same. Figure 10 is the error curves. The red curve is the error between the prediction value and the true value of the GUR when the number of nodes in the hidden layer is 11. The blue line curve is the error between the prediction value and the true value of the GUR when the hidden layer node is 7. It can be seen from Figure 10 that the training error is much smaller when the node number is 11, so we choose 11 as the number of hidden layer nodes in this paper. 

**Figure 11:**
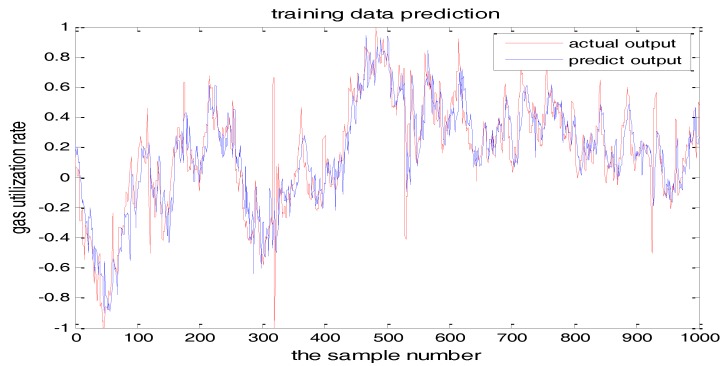
The TS-FNN training result when the central parameter *c* = 0.5.

**Figure 12:**
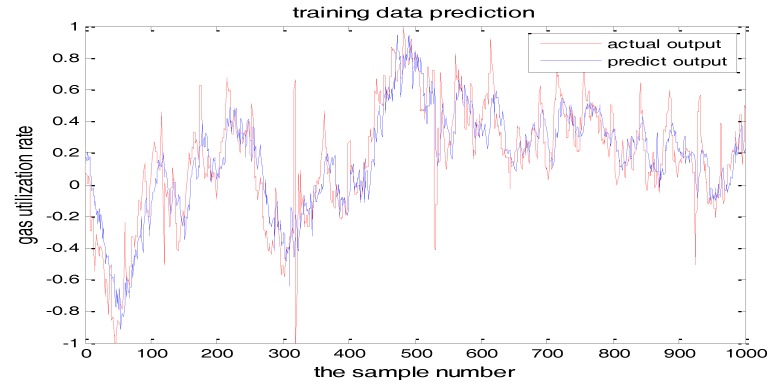
The TS-FNN training result when the central parameter *c* = 0.05.

**Figure 13:**
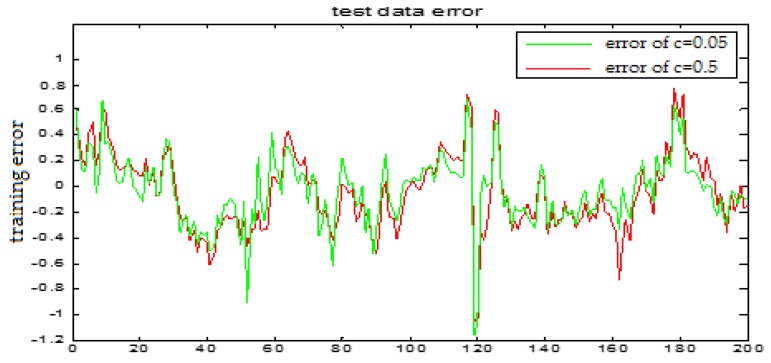
The TS-FNN training error curve of the central parameter *c* = 0.5 and *c* = 0.05 respectively.

The selection of the central parameters of TS-FNN also has a big influence on the output. Figure 11 and Figure 12 show the prediction results when the central parameter *c* = 0.5 and *c* = 0.05, respectively while all the other parameters are the same. Figure 13 is the error curve in these two cases in which the red curve is the results when *c* = 0.5, and the green curve is the results when *c* = 0.05. It can be seen from the figures that the training error of *c* = 0.5 is much smaller. 

**Figure 14:**
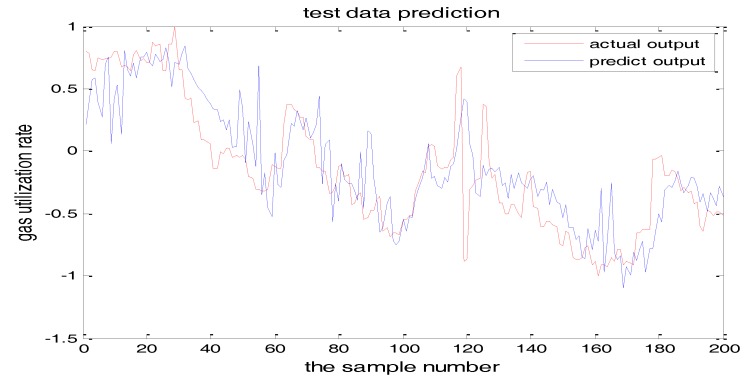
The TS-FNN testing result when the number of hidden nodes is 11.

**Figure 15:**
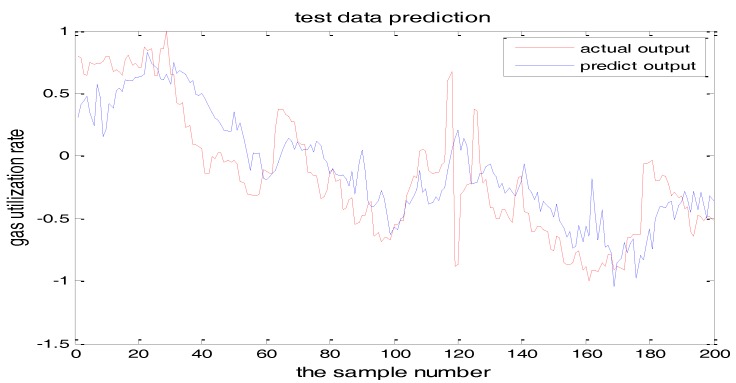
The TS-FNN testing result when the number of hidden nodes is 7.

**Figure 16:**
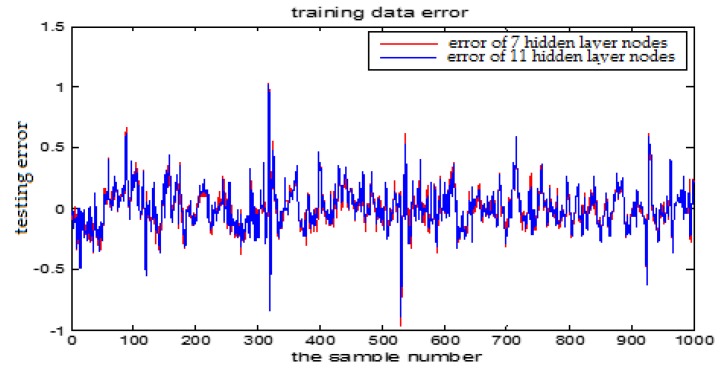
The TS-FNN testing error curve of 7 and 11 hidden layer nodes.

Figure 14 and Figure 15 show the training error when the number of hidden nodes is 11 and 7, and the other parameters are the same. Figure 16 is the error curve in two cases, in which the red curve is the error when the number of the hidden layer nodes is 7. The blue curve is the error when the hidden layer node number is 11. It can be seen from the graph that the prediction error is very small when the node number is 11.

**Figure 17:**
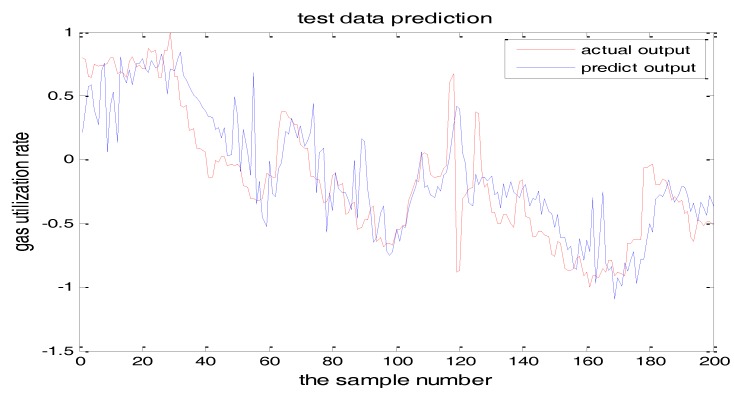
The TS-FNN testing result when the central parameter *c* = 0.5.

**Figure 18:**
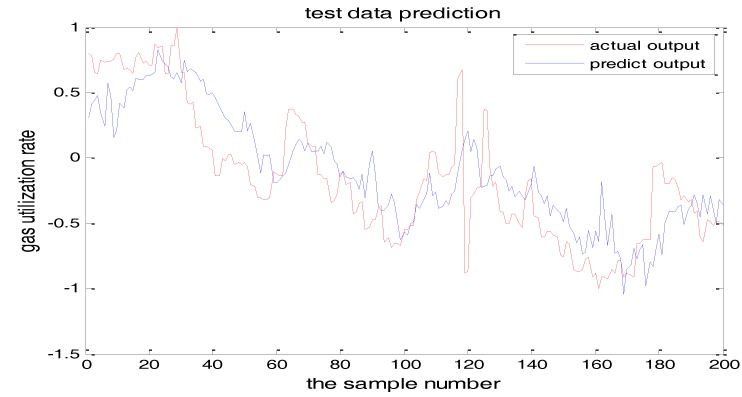
TS-FNN testing result when the central parameter *c* = 0.05.

**Figure 19:**
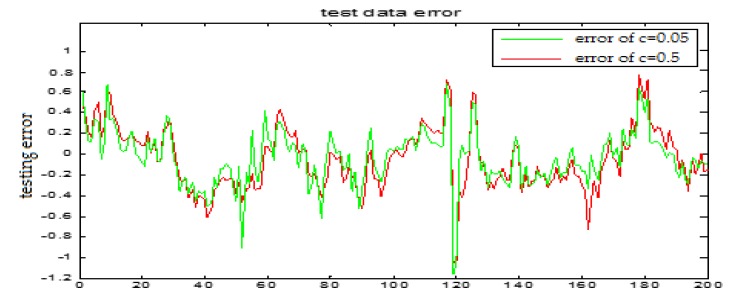
TS-FNN testing error curve of the central parameters *c* = 0.5 and *c* = 0.05, respectively.

Similarly, Figure 17 and Figure 18 are the testing results when the central parameters are *c* = 0.5 and *c* = 0.05 while the other parameters are the same. Figure 19 is the error curve in two cases, in which the red curve is the prediction error curve when *c* = 0.5, and the green curve is the prediction error curve when *c* = 0.05. It can be seen from the figures that the testing error when *c* = 0.5 is much smaller.

From the above figures, we reach the following conclusions: when the hidden layer node is 11, the training error and the testing error is smaller than the error when the hidden layer node is 7. The results will be better when the center parameter is *c* = 0.5. Therefore, the hidden layer nodes selected in this paper is 11 and the center parameter *c* is selected as 0.5.

## 5. The Proposed PSO-TS-FNN Algorithm

### 5.1. The Particle Swarm Optimization Algorithm 

Particle swarm optimization (PSO) [19,20,21] was proposed in 1995. It is an intelligent algorithm based on a simple social model. The theoretical foundation of the model is the social behavior of birds and fish in Nature. When the PSO algorithm is used to optimize neural network models, the principle is to treat every solution of the problem as the position of a bird in the search space, and we call them particles. In this paper, the specific meaning of the particle is the difference value between the predicted value and the expected value of the network. Each particle has a fitness value and a speed. The fitness is obtained by the optimization function. The role of speed is to determine the direction and distance of the particle’s flight. In solving the optimization problem, each particle follows one of the current optimal particles and searches in the solution space. 

The process of PSO is to initialize a group of particles firstly. The initialization process is random. Then PSO searches for the optimal solution by iteration. It should be noted that at each iteration, the particles update their position through the pursuit of the two kinds of particles. One is called pbest which is the optimal solution of the particles. The other one is called gbest which is the optimal solution of the whole group. There are *n* particles and D dimensional searching spaces and their mathematical expressions are as follows:

(15) $$V_{id}(t+1)=\omega^{0}V_{id}t+c_{1}r_{1}\left(P_{id}-X_{id}\left(t\right)\right)+c_{2}r_{2}\left(P_{gd}-X_{id}\left(t\right)\right)$$

(16) $$X_{id}\left(t+1\right)=X_{id}\left(t\right)+V_{id}(t+1)$$

where, $X_{i}=\left(X_{i1},X_{i2},\cdots ,X_{id}\right)$, $V_{i}=(V_{i1},V_{i2},\cdots ,V_{id})$ are the current position and the current flight speed of the particle. $P_{i}=\left(P_{i1},P_{i2},\cdots ,P_{id}\right)$, is pbest mentioned above, which represents the optimal position of the current particle. Pg=(Pg1,Pg2,⋯,Pgd) is gbest, it represents the optimal location of the entire particle swarm. ω is the inertia factor. It is a non-negative constant. $c_{1},\;c_{2}$ are the learning factors. It is also a nonnegative constant. $r_{1},\;r_{2}$ is randomly generated. The range of them is from 0 to 1. $V_{id}\in \left[-V_{max},\;V_{max}\right]$, $V_{max}$ is the maximum rate of the current particle. It is the number of iterations at present. 

### 5.2. Particle Swarm Optimization (PSO) Fuzzy Neural Network of TS Model 

**Figure 20:**
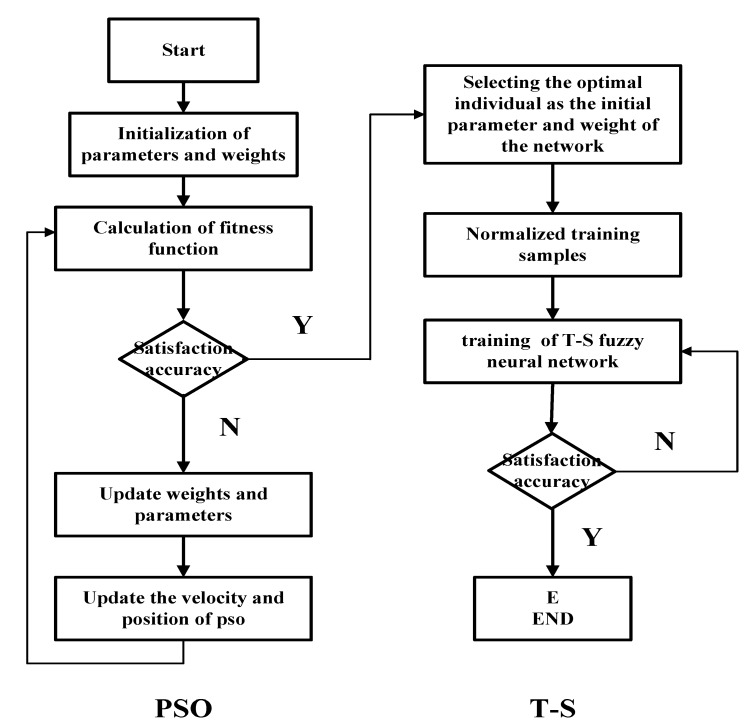
PSO+T-S algorithm flowchart.

The central parameters C and the bandwidth B of the TS-FNN are randomly generated according to the number of the nodes in the input layer and the number of nodes in the hidden layer. They are adjusted according to the difference between the actual output and the predicted output of the model. The particle swarm optimization [22,23] method can optimize the selection of the parameters C and B to improve the precision of the model. In Figure 20, we show the flow chart of the particle swarm which is to optimize the parameters C and B of the T-S fuzzy model. The steps of the particle swarm optimizing the parameters C and B of the T-S fuzzy model are as follows [24]:

Step 1: Initialization. Initialize the parameters and the weights.
Step 2: Modeling. Establish the T-S model and calculate the learning algorithm weights based on the output error. The training error is used as the Fitness function of the particle swarm.
Step 3: Update. Update the parameters and the weights. Then update the particle’s velocity and its position.
Step 4: Judgment. Determine whether the number of iterations of the particle swarm reaches the maximum. If so, calculate the optimal parameters of the T-S model. If not, go back to Step 3.
Step 5: The Second Judgment. Determines whether the iterations of the T-S model have reached the maximum value. If so, the modeling is over. Otherwise, go back to Step 2.

**Figure 21:**
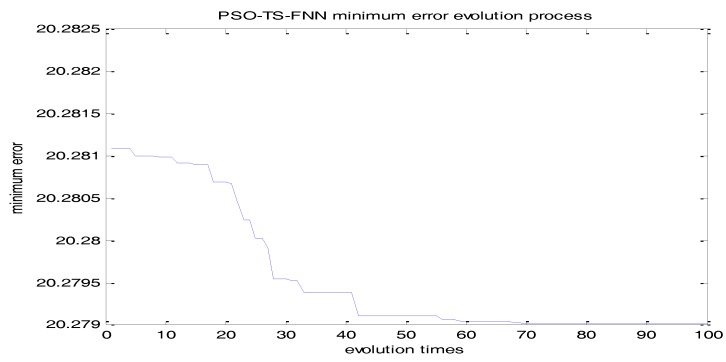
Fitness curve of particle swarm.

In this paper, the particle refers to the difference between actual output and the predicted output. According to the simulations tests, the initialized particle swarm size is selected as 40. The number of the input layer and the hidden layer determines the number of particles (110 particles.). Learning factors C1 and C2 are set to 1.8. The velocity is updated in the range of {−30, 30}. The speed is updated in the range of {0.0001, 10}. The maximum evolution number of the particle swarm is 100 according to the fitness change curve of the particle swarm in Figure 21. It can be seen from the figure that the PSO converges after 76 iterations. 

**Figure 22:**
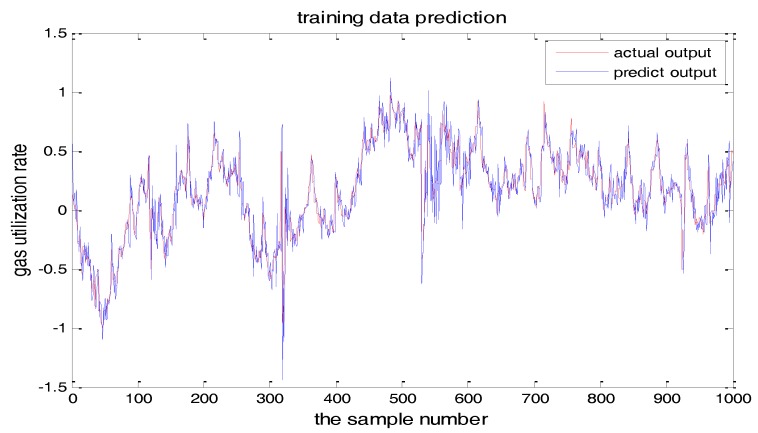
PSO+TS-FNN training result

**Figure 23:**
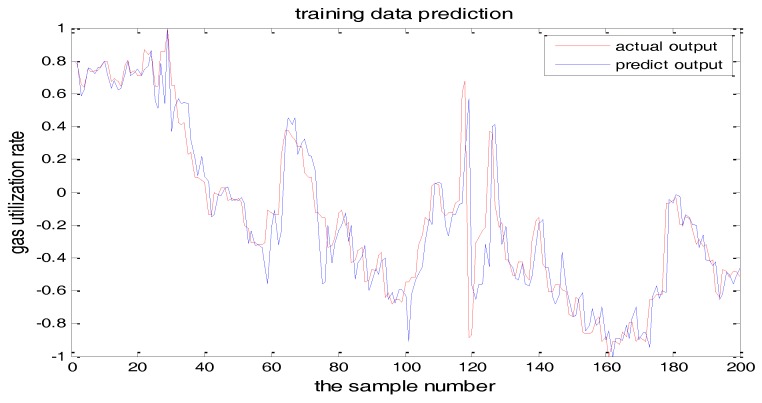
PSO+TS-FNN testing result.

PSO-TS-FNN training results were shown in Figure 22 and Figure 23 showed the testing result. It can be seen from the figures that the prediction effect of the particle swarm optimized model has good prediction accuracy. 

## 6. The Performance Comparison of Different Models 

**Figure 24:**
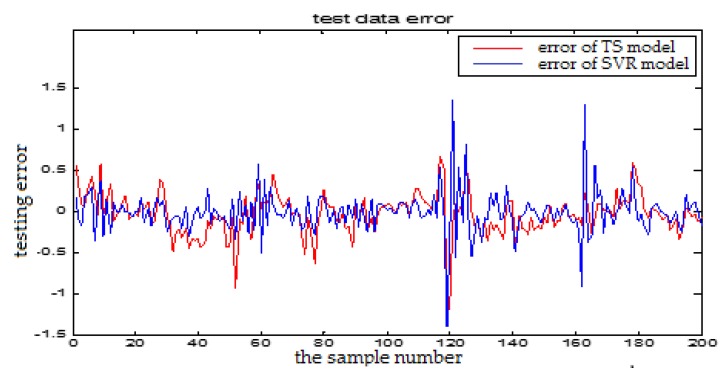
The testing error curve of TS-FNN model and SVR model.

**Figure 25:**
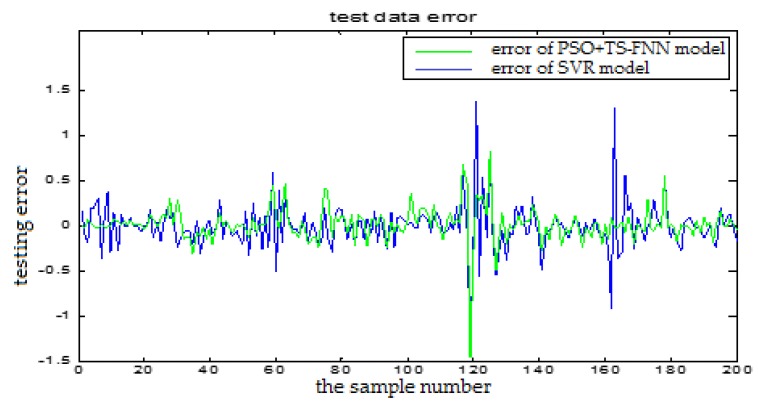
The testing error curve of SVR model and PSO+TS-FNN model.

In this section, we will show the prediction performance comparison which is represented by the error figures. One is the error curve between the predictive output and the actual output of the TS-FNN model. The other one is the error between the predictive output and the actual output of the PSO+TS-FNN model. In the following, the proposed algorithm is also compared with the SVM based model. In Figure 24, the blue curve represents the prediction error of the SVR model. The red curve is the prediction error of a single TS model. In Figure 25, the green curve is the prediction error of the TS model optimized by PSO. From the error graph, it can be seen that the particle swarm optimization model is better than the single TS model and the SVM-based model.

To further compare the TS fuzzy neural network’s predictive performance before and after optimization, we also calculated the average relative error [25] and the root mean square error [26] of the prediction value and the actual value. The calculation formula can be seen in Equations (17) and (18):

(17) $$AEA=\frac{1}{n}\overset{n}{\underset{i=1}{\sum }}\left|yi^{\prime }-yi\right|$$

(18) $$MSE=\frac{1}{n}\overset{n}{\underset{i=1}{\sum }}\sqrt{{\left(yi^{\prime }-yi\right)}^{2}}$$

where *n* is the number of the samples, yi is the actual value of GUR, and $yi^{\prime }$ is the prediction value from the model output. 

The analysis table (Table 2) showed that the root mean square error of the SVR model is 0.0679 and the test error of the proposed model with the particle swarm optimization is 0.0460. From the table, we can see that the proposed optimized model has better prediction performance. The proposed method can better meet the actual needs of the blast furnace production. 

## 7. Conclusions

At present, the research on GUR is mainly concentrated on the mechanism aspects. The complex working environment of the blast furnace determines that the mechanism model cannot accurately describe the strong nonlinear relationship between the GUR and the operation parameters. To solve this problem, a TS-FNN based on the PSO is proposed to predict the GUR. Through the analysis of the mechanism level, we preliminarily proposed the approach on how to choose the input parameters of the model and how to analyze the correlation between the related parameters and the GUR through the method of mutual information. In order to ensure the accuracy of the model, the selected inputs were de-noised to remove the abnormal values. Finally, the TS-FNN is applied to the model, and PSO is used to optimize the bandwidth parameters and the central parameters of the optimization model. The experimental results show that this method can accurately predict the GUR. The paper compared the proposed method with TS-FNN without PSO and the SVM method. The proposed method gave a MSE error of 0.046 while the TS-FNN without PSO method has a MSE error of 0.0599, SVM model has a MSE error of 0.0679. The proposed method thus has better performance than the other two methods. All of them can predict the GUR. SVM has less parameters to be estimated. The proposed algorithm has moderate speed. It can provide strong theoretical support for the optimization of the operation and the energy saving of the BF.

## Figures and Tables

**Table 1:** The correlation results of the operation parameters and GUR.

Operating Parameters	Mutual Information
Blast volume	0.6770
Blast pressure	0.6820
Top pressure	0.6253
Top temperature	0.9455
Oxygen enrichment	0.6732
Gas utilization ratio	2.9184

**Table 2:** The error of different models.

Algorithm Model	AEA		MSE	
	Training	Testing	Training	Testing
SVR	0.2661	0.3856	0.0297	0.0679
FNN	0.1284	0.3064	0.0265	0.0599
PSO-T-S FNN	0.0894	0.2132	0.0210	0.0460

## Appendix A

**Table A1:** The abbreviations used in this paper.

Abbreviation	Explanation
GUR	Gas utilization ratio
BF	blast furnace
FNN	fuzzy neural network
TS-FNN	TS fuzzy neural network
PSO	particle swarm algorithm
PSO-TS-FNN	particle swarm algorithm (PSO) to optimize the TS-FNN
FW	blast temperature
FY	blast pressure
DY	top pressure
DW	top temperature
O2	enrichment percentage
MQLYL	GUR
PCC	Pearson correlation coefficient
SCC	Spearman correlation coefficient
